# Public healthcare as a destroyer of value – a customer perspective

**DOI:** 10.1108/JHOM-05-2025-0233

**Published:** 2025-11-04

**Authors:** Petra Hurme

**Affiliations:** Faculty of Management and Business, Tampere University, Tampere, Finland

**Keywords:** Value destruction, Value co-destruction, Customer value, Public healthcare, Lean management

## Abstract

**Purpose:**

This study explores how customer value is destroyed in Lean-managed public healthcare by applying the concepts of value destruction and value co-destruction.

**Design/methodology/approach:**

The study draws on a qualitative analysis of customer feedback (*n* = 507) collected in 2024 from general practitioner and nurse consultations and oral healthcare services, including telephone interactions related to these services, within a Finnish wellbeing services county operating under Lean management principles. The dataset was limited to responses with a Net Promoter Score of 0, accompanied by open-ended comments detailing reasons for perceived value loss. An abductive content analysis was conducted, guided by existing theories of value destruction and co-destruction.

**Findings:**

Customer value was destroyed or co-destroyed due to restricted access to care, dysfunctional service processes and inadequate service encounters. Some mechanisms reflected systemic failures that excluded customers from interaction altogether, while others emerged during deteriorated service encounters. These issues resulted in confusion, compromised self-care, emotional harm and a breakdown of trust.

**Originality/value:**

This study contributes to service research by operationalising the concepts of value destruction and value co-destruction using empirical data from a Lean-managed public healthcare organisation. It challenges the assumptions of public service logic, which positions value co-creation as a normative ideal, by demonstrating that co-creation may become structurally impossible when essential conditions such as service accessibility, continuity of care and professional communication are absent.

## 1. Introduction

The creation of customer value is a key objective in public organisations (Cui and Aulton, 2023; Osborne *et al.*, 2021; Virtanen and Jalonen, 2024). However, achieving this goal can be particularly challenging when customer needs are more diverse than the structure and capacity of the public service system can accommodate (Gyllenhammar *et al.*, 2023). Nonetheless, public services have the potential to operate in a user-oriented and efficient manner, as long as they are supported by effective service management, forward-thinking professionals, and a commitment to reforming outdated structures (Grönroos, 2019).

Value is both created and destroyed at all levels of public service activity (Skarli, 2023). Value creation and destruction are closely interconnected and can at times form part of the same interaction process (Echeverri and Skålén, 2021). Value, and its deterioration, is a complex phenomenon that must be examined from the perspectives of multiple actors, as the creation, deterioration or loss of one form of value can directly influence others (Naveed *et al.*, 2025).

Value destruction refers to a deterioration of customer value caused by structural barriers, systemic failures or exclusion from service processes, occurring in the absence of interaction between the customer and the service provider (Cui and Osborne, 2023; Singhvi *et al.*, 2016). In contrast, value co-destruction tends to arise when customer-provider interactions are misaligned, ineffective or characterised by conflicting expectations (Lumivalo *et al*., 2024).

The core assumption of public service logic (PSL) is that value is not created by organisations alone, but rather emerges through the needs, experiences and involvement of service users (Cui and Aulton, 2023; Virtanen and Jalonen, 2024), as well as through their interaction with service providers (Eriksson, 2019; Gyllenhammar *et al.*, 2023). However, its applicability may be limited in contexts where basic services are inaccessible or inconsistent, and it risks reinforcing inequality, as not all customers are equally able to participate in co-creation processes (Kinder and Stenvall, 2023). This perspective may overlook situations in which co-creation is not possible, due to structural or systemic barriers that prevent interaction altogether. In such cases, value is not co-destroyed but destroyed before any interaction can occur.

This study examines how customer value is destroyed in Lean-managed public healthcare, focusing on mechanisms reported by service users. Rather than treating value destruction and co-destruction as strictly separate categories, the study applies both concepts to explore a broader spectrum of value-destroying processes, including those that occur without any customer involvement. Both concepts are incorporated into the analysis to capture the full spectrum of value loss as expressed in customer feedback. Instead of drawing a strict conceptual boundary between destruction and co-destruction, the study investigates how customers experience value deterioration as arising from absence of contact, systemic breakdowns, or distorted interaction.

The study was conducted in a public wellbeing services county in Finland, committed to Lean management, where the central aim is to create customer value (Ballé and Jones, 2017). Lean is widely applied in healthcare (Leite *et al.*, 2024; Winner *et al.*, 2022), as it aims to eliminate non-value-adding steps by organising process functions from the perspective of the value created for patients (Ojo *et al.*, 2022). Service processes are often too complex to be fully governed by Lean principles alone and may require complementary logics such as public service logic (Singhvi *et al.*, 2016). However, as this study shows, even this combination may prove insufficient when basic conditions for interaction are not met.

This article is based on qualitative research, drawing on customer feedback (*n* = 507) collected in 2024 from one Finnish wellbeing services county, covering consultations with general practitioners and nurses, oral healthcare services, and telephone service contacts. The Net Promoter Score (NPS) is an internationally used customer loyalty metric that measures the likelihood of recommending a service on a scale from 0 to 10. The data was limited to responses where the NPS was rated 0, accompanied by written feedback describing the customer’s experience of value destruction. The research question was: What factors destroy value in Lean-managed public healthcare services from the customer’s perspective? In this study, the term customer is used broadly to refer to users of public healthcare services as well as residents who are affected by these services. Value is understood as a subjective experience in relation to an individual’s personal wellbeing (Cui and Aulton, 2023). Based on the findings, it can be concluded that barriers to accessing care, dysfunctional care processes, and problems in service encounters are the most significant factors contributing to the destruction of customer value.

While both Lean and public service logic research have predominantly focused on value creation and co-creation, the mechanisms through which value is destroyed or co-destroyed have received significantly less attention (Engen *et al.*, 2021; Eriksson and Nordgren, 2018; Leite and Quadros, 2024; Naveed *et al.*, 2025). While customer participation in Lean development has been shown to provide collective benefits from a management perspective, only a few studies specifically address the customer’s viewpoint (Fernandes *et al.*, 2020; Gray *et al.*, 2023). This study addresses these gaps by examining how customer value deteriorates in Lean-managed public healthcare from the viewpoint of customers.

## 2. Theoretical framework

### 2.1 Value and customer value

Value is an inherently complex and contested concept, particularly in public service contexts where multiple actors, competing priorities, and normative expectations intersect (Dudau *et al.*, 2019; Höglund *et al.*, 2021; Ng and Smith, 2012; Voorberg *et al.*, 2015). In the public sector, value can take both individual and collective forms, which may align or conflict (Osborne, 2018). Compared to the private sector, public value is further complicated by considerations of fairness, equality and long-term wellbeing (Alford, 2016; Kinder and Stenvall, 2023; Naveed *et al.*, 2025). In healthcare, value often emerges over time and across multiple service interactions, adding further complexity to its definition (Keeling *et al.*, 2021).

Customer value, then, is not a fixed property of a service but a contextual and subjective experience that depends on the customer’s situation and expectations (Komulainen *et al.*, 2023; Strokosch and Osborne, 2020). A clear understanding of the customer is essential, particularly in public healthcare where service structures may not align with individual needs (Cheng *et al.*, 2015; Poksinska, 2010). In this study, customer value is understood as a subjective experience in relation to personal wellbeing, emerging through or being obstructed by interactions with the public healthcare system (Cui and Aulton, 2023). While the term “customer” refers to a user of public services or someone seeking access to care, it more broadly encompasses any individual affected by service-related interactions (Chandler and Vargo, 2011; Gyllenhammar *et al.*, 2023).

### 2.2 Value destruction

Value destruction refers to the deterioration or loss of value resulting from systemic failures, structural constraints, or the complete absence of meaningful interaction between the customer and the service provider (Cui and Osborne, 2023; Singhvi *et al.*, 2016). In public healthcare, value is not always created: it may be actively undermined, particularly when services are inaccessible or fragmented (Cui and Osborne, 2023a). Value destruction may occur independently of customer-provider interaction, for example when the user is unable to access services at all, but it may also co-exist with value creation, diminishing the benefits a service might otherwise deliver (Singhvi *et al.*, 2016).

While the concept remains relatively underdeveloped and context-dependent (Abid *et al.*, 2022), it offers a lens to analyse the breakdown of value within public systems that are expected to promote equity and wellbeing. In this study, value destruction is not treated as conceptually separate from co-destruction, but as part of a continuum of value loss. The term is used to capture customer experiences in which value was prevented from emerging altogether, particularly due to dysfunctional structures, service bottlenecks, or systemic exclusion (Leite and Quadros, 2024).

### 2.3 Public service logic and value Co-destruction

Public Service Logic (PSL) challenges the traditional notion that public organisations create value through service delivery. Instead, it conceptualises value as emerging in use – through the customer’s lived experience – while positioning the organisation as a facilitator (Alford, 2016; Osborne, 2018). This view highlights the centrality of interaction, emphasising that value co-creation is only possible when the user can actively engage with accessible, responsive, and well-functioning services (Naveed *et al.*, 2025; Skarli, 2023).

However, PSL has been criticised for assuming that customers are always able and willing to participate in co-creation. In practice, structural barriers, service fragmentation, and resource constraints can prevent users from engaging meaningfully with services (Kinder and Stenvall, 2023). In such cases, co-creation is not just inhibited – it becomes structurally impossible. This study draws on these critiques to explore how the assumptions of PSL may not hold in contexts where even minimal interaction is absent.

Recent research has extended PSL by examining value co-destruction, which occurs when misaligned interactions, insufficient support, or conflicting expectations lead to negative outcomes for customers or service systems (Leite and Quadros, 2024; Lumivalo *et al.*, 2024). Co-destruction can result from either organisational shortcomings or customer-related factors, such as lack of capacity to engage with complex services (Engen *et al.*, 2021). In healthcare, these dynamics unfold at a sensitive interface, where co-creation can quickly shift into co-destruction unless supported by adequate institutional conditions (Skarli, 2023).

## 3. Lean management as the context of the study

Lean is a coaching-oriented management approach aimed at increasing value through continuous improvement and a comprehensive cultural transformation (Ballé *et al.*, 2019; Cohen, 2018), with value being defined by the customer (Fernandes *et al.*, 2020; Ojo *et al.*, 2022). Although Lean engages frontline healthcare professionals in improving processes (Cohen, 2018), sustainable value creation also requires customer involvement (Hurme and Liljeroos-Cork, 2024). Successful implementation of Lean necessitates a change in management approach at all organisational levels (Kahm and Ingelsson, 2020). In particular, the commitment of top management and political decision-makers is crucial (Camuffo and Poletto, 2024). A holistic understanding of the organisation’s operations promotes the identification of disruptions in value streams (Bhasin and Found, 2021).

Lean is utilised in the healthcare sector to address recognised problems (Korhonen *et al.*, 2016; Leite *et al.*, 2024; Winner *et al.*, 2022), and the way in which it is implemented varies significantly between organisations (Reponen and Torkki, 2022). Typically, Lean in healthcare has focused on continuous improvement, the creation of customer value, respect for people, and the efficient use of resources (Maijala *et al.*, 2020). Although Lean has succeeded in creating value (Lima *et al.*, 2021), it has also resulted in value destruction (Leite and Quadros, 2024), particularly when there is an excessive focus on internal organisational processes (Chen *et al.*, 2024; McAdam *et al.*, 2022). Conversely, focusing solely on customers does not necessarily yield the desired outcomes (Radnor and Osborne, 2013). At its best, Lean promotes internal organisational value, such as learning, an emotionally healthy and safe working environment, autonomy, and clear structures (Fenner *et al.*, 2023; Sales and De Castro, 2021), thereby strengthening customer involvement and customer value (Radnor and Osborne, 2013). At its weakest, Lean results in failures and only short-term outcomes (Lindsay *et al.*, 2020). However, it remains uncertain whether the outcomes are truly attributable to Lean, or rather to the leader’s own approach and choices in managing change (Pedersen and Huniche, 2011).

## 4. Lean in the case organisation

The case organisation is a Finnish wellbeing services county responsible for organising public healthcare, operating under a universal, tax-funded national system (Keskimaki *et al.*, 2019).

In the Finnish wellbeing services county where this study was conducted, Lean was officially adopted at the beginning of 2019, when the region transitioned to a joint municipal authority model in anticipation of the national shift to wellbeing services counties. Lean was introduced with the explicit aim of improving service quality and standardising processes across the newly formed organisation. It was seen as a way to harmonise and enhance operational practices while creating a culture of continuous improvement. Clear goals and performance indicators were set for the implementation of Lean, reflecting a strategic commitment to embedding Lean principles into the management system. While internal metrics and performance indicators guided the deployment of Lean, this study focuses specifically on how customers experienced value loss within that system. The Roidu customer feedback system was not directly linked to the implementation of Lean but supports its objectives by providing measurable data on customer satisfaction.

## 5. Methodology

### 5.1 Data collection

The data for this qualitative study were collected in 2024 from a total of 507 customer feedback responses submitted via the electronic Roidu system of a Finnish wellbeing services county. The feedback covered consultations with general practitioners and nurses, oral healthcare services, and telephone services associated with these functions.

The Roidu system offers multiple feedback channels: a link sent automatically via text message following all telephone service contacts, feedback terminals at physical service points, and an open feedback form on the organisation’s website. While the SMS channel was only available to individuals who had actively contacted services, the other channels were publicly accessible. As a result, the exact identity of respondents cannot be verified, and it is possible that some feedback was submitted by caregivers or other residents. However, since all inhabitants of the wellbeing services county are potential users of public healthcare services, the term “customer” is used in a broad and inclusive sense throughout the study.

For the purpose of this study, only responses that included both a Net Promoter Score (NPS) rating of 0 (the lowest possible) and a written comment were selected. In particular, only those comments that provided a clear explanation for the dissatisfaction were included; vague or purely emotional feedback without specific content was excluded from the analysis. No biographical or personally identifiable data were collected.

The research was conducted with the permission of the wellbeing services county, which is committed to Lean management. The organisation saw the study as an opportunity to better understand customer experiences and identify potential barriers to value creation.

### 5.2 Data analysis

Open-ended customer feedback was analysed using abductive content analysis to gain a deeper understanding of the mechanisms of value destruction. Theoretical concepts of value destruction and co-destruction guided the analytical process: they were used to refine data-driven codes into broader categories and final themes through iterative comparison. The analysis was conducted by a single researcher, with Atlas.ti software used to support the organisation and structuring of the data. The material was reviewed multiple times to ensure in-depth familiarity with its content.

The coding process was iterative, involving constant comparison and abstraction. Similar codes were grouped into conceptual categories, which were then synthesised into higher-level themes. The analysis continued until thematic saturation was reached—meaning no new significant patterns emerged from the data. The abductive approach enabled a dialogue between theory and empirical material, which informed the interpretations (Saldana *et al.*, 2011, 93). To preserve anonymity, certain words and references were removed from quotations where they could reveal specific service locations or regions, without altering the substantive meaning of the feedback.

## 6. Results

The findings of the study indicate that customers may experience value destruction at all stages of healthcare service processes. Value destruction occurs during different processes but also in between them. Customers reported value being destroyed due to barriers to accessing care, dysfunctional care processes and problems in encounters. Value destruction was experienced as uncertainty, concrete difficulties in self-care, and a sense of worthlessness and abandonment.

### 6.1 Barriers to accessing care

These findings illustrate value destruction caused by structural barriers to access. Care was inaccessible even before any interaction took place (Cui and Osborne, 2023).

#### 6.1.1 Systemic resistance

According to the results, customers felt that they needed to know how to demand access to care and even lie about symptoms and their condition in order to receive treatment. Being stigmatised due to obesity, mental health issues, or ethnicity was experienced as a barrier to care. Booking appointments was perceived as impossible due to data protection restrictions. Feedback also highlighted that certain ailments were not considered worthy of treatment in the public sector, whereas in the private sector the opposite was true.

A person should not be categorised because of past issues or mental health problems, somatic symptoms must be investigated even if labelled crazy. Everyone knows their own body!!

#### 6.1.2 Appointment booking process

The appointment booking process was considered challenging in several ways. It was regarded as problematic that appointments had to be made by phone, with no electronically bookable times available. Furthermore, the operation of the telephone service was seen as difficult, as call-backs or other contact were slow or, in some cases, never occurred. The digital needs assessment tool in use was experienced as confusing, generating follow-up contacts for issues not reported by the customer. Rescheduling appointments was perceived as inflexible, and notifications about cancellations were not received.

#### 6.1.3 Queues and delays

Queues and delays in accessing care were seen as a significant value-destroying factor. Generally, customers felt that getting an appointment was difficult and slow. Serious illnesses were not taken into account in waiting times, and even children faced unreasonably long waits for care. A bureaucratic approach to the customer’s issue also caused significant delays or entirely prevented access to care.

Getting to a dentist for a check-up is a distant hope. So I decided to go private. After that, I tried to book an appointment with a dental hygienist, but it was impossible because you first have to visit a municipal dentist for a check-up. Absolutely ridiculous. I saved municipal funds and shortened the queue by going private and now I can’t see the hygienist unless I go for another check-up.

#### 6.1.4 Challenges for working people

Especially working customers experienced barriers in booking appointments and accessing care. They reported that using the telephone service was not feasible, as responding to call-backs was often impossible during working hours. Attending appointments during the workday was also considered challenging, and evening appointments were not available. In addition, walk-in services required waiting for hours without knowing when one would be seen, which was difficult to manage during the working day.

My child was supposed to have a 20-minute appointment, and I was required to take time off work to transport them. The appointment was 20 minutes late and lasted significantly longer than scheduled. The entire class’s parents were there at the same time, and we were all using our work time for transport. If you schedule school children’s check-ups during the day, the timing must be accurate.

### 6.2 Dysfunctional care processes

These findings illustrate value destruction that occurs within public healthcare organizational processes due to delays, errors, and discontinuities in care. Such breakdowns in organisational functioning reduce the value delivered and reflect the kinds of process failures discussed by Singhvi *et al.* (2016) and Cui and Osborne (2023).

#### 6.2.1 Delays or postponement of treatment

Delays in appointments were experienced as challenging, particularly in the case of child patients. Customers with multiple concerns could not address all issues in one visit, causing further delays. Some reported repeated cancellations and postponements of appointments, at worst leading to the loss of sickness benefits. Failure to renew prescriptions resulted in customers running out of medication and being unable to continue treatment. Some issues required multiple contacts to resolve.

I received a doctor’s certificate from the hospital. The subsequent sick leave certificates had gaps. Through my nurse, I got a certificate and an appointment only after incurring double self-liability time. Financially, this meant a €180 loss in sickness benefits.

#### 6.2.2 Errors

Customers reported various errors that contributed to value destruction during the treatment process. These included the disappearance of medical records when they were needed for care, missing entries in the Oma Kanta system, and lost laboratory results. In addition, customers gave feedback about incorrect diagnoses being recorded in medical records, wrong medications being prescribed, and inappropriate treatment being administered. Further concerns involved the provision of inaccurate information, inadequate pain management during appointments, and situations where the parents of minors were not informed about procedures or asked for their or the child’s opinion.

Breaches of data protection emerged in various ways. Customers reported professionals discussing other patients’ matters loudly and in public, or leaving patient information visible in consultation rooms. A proxy form containing personal data was reported lost and never recovered. Text messages intended for other patients were also said to have been sent to the wrong recipients.

At no point was the guardian’s or the child’s opinion asked. The procedure was entirely unnecessary, and they could have simply advised us to let the tooth loosen naturally at home. At the very least, they should have checked if it was acceptable to us.

We stayed in the room with my child waiting, and the computer screen showed not only my child’s data but also at least five other patients’ information, including personal identity numbers. The screen had no privacy protection; all data was visible to us for several minutes.

#### 6.2.3 Staff turnover

The frequent turnover of professionals was highlighted in situations where customers had multiple appointments within a short period. Customers found it frustrating to repeat the same issues over and over again to constantly changing professionals. They felt that with each new contact, the matter had to be started from the beginning. Continuous staff turnover disrupted the continuity of care and contributed to confusion within care processes.

### 6.3 Problems in service encounters

These findings demonstrate how value co-destruction can occur in direct service encounters between professionals and customers. Poor interaction, whether due to language barriers or dismissive behaviour, undermines trust and customer wellbeing. Even brief contact can turn value creation into value loss when respect and understanding are lacking (Echeverri and Skålén, 2021; Engen *et al.*, 2021).

#### 6.3.1 Language barriers

Customers reported situations where they could not understand the professional due to the latter’s limited proficiency in Finnish. As a result, they felt their concerns were not heard or understood. Some customers wished to receive services in English, but the professional’s language skills were not sufficient to ensure their issue was properly understood and addressed.

#### 6.3.2 Behaviour of professionals

The behaviour of professionals was a significant factor in value destruction. Customers felt that professionals did not listen, were not interested, and showed no willingness to help. Feedback highlighted a lack of empathy, as well as reports of indifference and failure to acknowledge fear. Rude, arrogant, and dismissive attitudes were also mentioned. Customers described experiences of indifference and recounted witnessing swearing, snapping, raised voices, and generally irritated service.

Getting care is already difficult, so why make it harder with inappropriate behaviour?

### 6.4 Effects of value-destroying factors on the customer

These effects illustrate the consequences of both value destruction and co-destruction, as experienced and expressed by customers. They reflect the emotional and practical impact of systemic failures and harmful interactions.

#### 6.4.1 Uncertainty

Uncertainty related to both access to care and various stages of the care process. Uncertainty about accessing care stemmed from the telephone service, particularly regarding when a call-back might occur. Customers felt they had to remain constantly on standby in case of contact. There were many uncertainties about the timing of contact, as it often did not occur as agreed or sometimes never happened. Uncertainty also related to queues customers were placed in after initial contact. Often, they had no idea of the length of the queue and were unsure when they would receive treatment. Uncertainty was also experienced during care processes, for example, when treatment had begun but was not completed, and the customer was placed in a queue mid-treatment without knowing how the matter would proceed.

I had to go private because waiting in the public system would have taken too long, and there was no certainty how long.

#### 6.4.2 Difficulties in self-care

Barriers to accessing care and dysfunctional care processes created challenges for customers in various situations. Difficulties were particularly evident among customers with chronic illnesses who relied on regular use of different medical supplies. Although the need for such supplies was continuous, when a device broke, a replacement could not be obtained quickly. Long delays in receiving new equipment made self-care difficult or even impossible.

It cannot take 2 weeks to get a call back, and ordering/spare parts is far too slow and difficult. If medical supplies break before Christmas, you say I’ll get a new one in about 3 weeks. What?

#### 6.4.3 Sense of worthlessness and abandonment

Feelings of worthlessness and abandonment arose both during attempts to access care and throughout the care processes. Customers described negative experiences during care, often involving disrespectful treatment or hurtful remarks. Such encounters caused significant negative emotions among customers. They also reported experiencing feelings of shame and despair while trying to access care, particularly in situations where gaining access to services was perceived as impossible despite a clear need for care.

Might as well give up and die.

I left crying because they were really rude to me. I felt helpless and abandoned; they were not at all helpful or willing to assist.

#### 6.4.4 Distrust

Distrust was experienced towards the service system at various levels. The professional’s competence and behaviour were key factors contributing to this distrust. Additional causes included situations where promised actions were not fulfilled, and instances where customers felt left alone to manage their issues without professional support or assistance. Witnessing breaches of data protection — such as seeing or overhearing other customers’ confidential information — also contributed to the erosion of trust in the system.

I’m sorry, but I was promised a call at 3pm and nothing has happened. If you can’t trust someone’s word, then what can you trust?

## 7. Discussion and limitations

### 7.1 Interpretation of findings

This study explored the factors that destroy value in Lean-managed public healthcare from the customer’s perspective. The analysis drew on the concepts of value destruction and value co-destruction to examine how value either failed to emerge altogether or deteriorated during service interactions. These concepts provided a framework for understanding the different ways in which customers experienced value loss, depending on whether contact with the service provider was absent or distorted. The findings show that value can be destroyed through barriers to accessing care, dysfunctional care processes, and problems in service encounters. These, in turn, lead to uncertainty, difficulties in self-care, and feelings of abandonment. Value creation and destruction often occur within the same service experience and are shaped by how customers define what is valuable to them. Barriers, lack of transparency, and service failures represent value destruction, as they prevent value from emerging at all (Cui and Osborne, 2023; Singhvi *et al.*, 2016). In contrast, unfriendly or dismissive interaction reflects value co-destruction, where value potential breaks down during contact between the customer and professional (Echeverri and Skålén, 2021; Engen *et al.*, 2021). In such cases, value is lost not only due to structures but also due to failed collaboration.

### 7.2 Theoretical implications

The findings of this study challenge the assumptions of public service logic (PSL), which emphasises co-creation between service providers and users (Osborne *et al.*, 2021). These findings reveal a core theoretical limitation in Public Service Logic: it assumes that users are able to participate in co-creation, yet this assumption breaks down when basic service conditions such as accessibility, continuity and clear communication are missing (Kinder and Stenvall, 2023). In such cases, customers are effectively excluded from interaction with service professionals, leaving value creation or destruction solely dependent on organisational structures and processes.

Thus, this study highlights the foundational role of service functionality as a precondition for value co-creation (Engen *et al.*, 2021; Gyllenhammar *et al.*, 2023). Without addressing systemic barriers and failures, the ideals of co-creation remain unattainable. These findings contribute to existing discussions by demonstrating that public service logic requires operational reliability and accessibility to function as intended, particularly in public healthcare contexts (Kinder and Stenvall, 2023).

### 7.3 Practical implications

The findings highlight the need for healthcare organisations to actively address institutional and process-based shortcomings in order to mitigate value destruction. Regular use of customer feedback in continuous improvement, combined with training staff in empathetic and responsive communication, is an essential step to reduce the risk of value loss (Hurme and Liljeroos-Cork, 2024). Furthermore, Lean management practices must evolve beyond internal process efficiency to genuinely integrate customer perspectives into service development (Radnor and Osborne, 2013). A user-centred approach is a key foundation for the development of well-being services (Poblete *et al.*, 2023).

For policy-makers, drawing on user experiences can support better alignment of resources and actors in addressing actual healthcare needs. To maximise sustainable value, organisations could adopt a value destruction-oriented operational model that systematically identifies and eliminates elements that erode value. Focusing only on value creation is insufficient—inefficient or poorly designed processes rarely create genuine value and may instead perpetuate dysfunction (Radnor and Osborne, 2013).

### 7.4 Societal implications

The study raises concerns about the welfare state’s ability to ensure equal access and equitable treatment for all in public healthcare services. When basic healthcare services fail, value creation becomes selective, benefiting only those who are able to navigate the system effectively. This risks undermining the principle of universal and equitable public services, supporting previous observations that public service logic does not operate effectively when basic service conditions are unmet (Kinder and Stenvall, 2023).

Structural reforms are needed to restore trust and inclusivity in public healthcare provision, particularly by addressing the needs of marginalised and hard-to-reach groups (Eriksson, 2019; Eriksson and Nordgren, 2018). Public service organisations should focus not only on core operations but also on promoting inclusive forms of co-creation throughout the service system.

### 7.5 Limitations and further research

This study deliberately focused on customers who had experienced value destruction in public healthcare services and who rated the service with a Net Promoter Score of 0. In this way, the factors related specifically to value destruction from the customer’s perspective were brought to light. The results therefore do not represent the experiences of all customers but deepen the understanding of situations where the service fails to produce value.

The data was collected from a single wellbeing services county, which limits the generalisability of the results to other contexts. However, the findings provide important insights into the phenomenon of value destruction in public healthcare and offer direction for further research.

In future research, it would be important to examine value destruction not only from the customer’s perspective but also from the perspective of professionals, to better understand how service practices can be developed to prevent value loss in daily operations. Comparative studies between different wellbeing services counties could provide insights into structural and organisational factors that support or hinder equitable service provision. Moreover, studying the long-term effects of value destruction, such as its impact on customers’ health, wellbeing, and trust in public services, is essential to inform policy reforms and strengthen the societal inclusiveness and reliability of public healthcare.

## 8. Conclusion

This study demonstrates that public healthcare not only fails to generate customer value in certain situations, but may actively destroy it. Both value destruction and value co-destruction occur within Lean-managed public healthcare organisations. Value destruction takes place particularly when access to services is difficult or entirely blocked. In such cases, no customer value can emerge. This reflects the definition of value destruction in the literature: the absence of the necessary conditions for value to materialise. Value co-destruction, in contrast, becomes evident when services are accessed but processes or interactions between the customer and the professional fail. These failures may stem from poor communication, lack of empathy or conflicting expectations. In this study, such encounters were frequently described as dismissive, confusing or emotionally distressing. They caused harm, eroded trust and ultimately undermined the potential for value creation.

**Figure 1:**
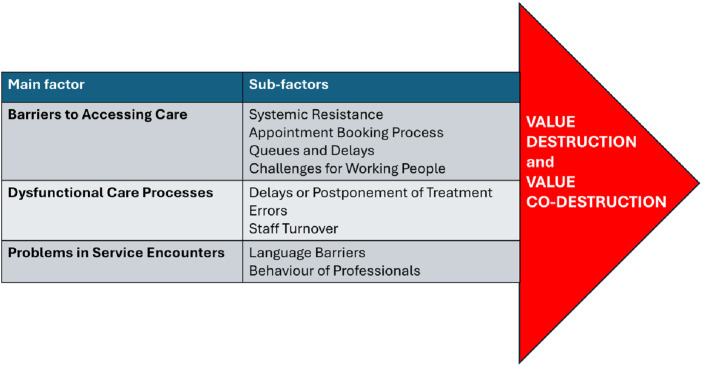
Main factors and sub-factors contributing to customer value destruction and co-destruction. Source: Author’s own work

According to public service logic, value is created through interaction between the service provider and the user. However, such interaction presupposes that services are both accessible and functional. Without these preconditions, customers are excluded from participation and value cannot be co-created. This raises a fundamental question: is public service logic a viable framework in contexts where users are unable to interact with services at all? If interaction does not occur, co-creation cannot take place. In such circumstances, the logic describes an idealised model rather than an empirical reality (see Figure 1).

**Figure 2:**
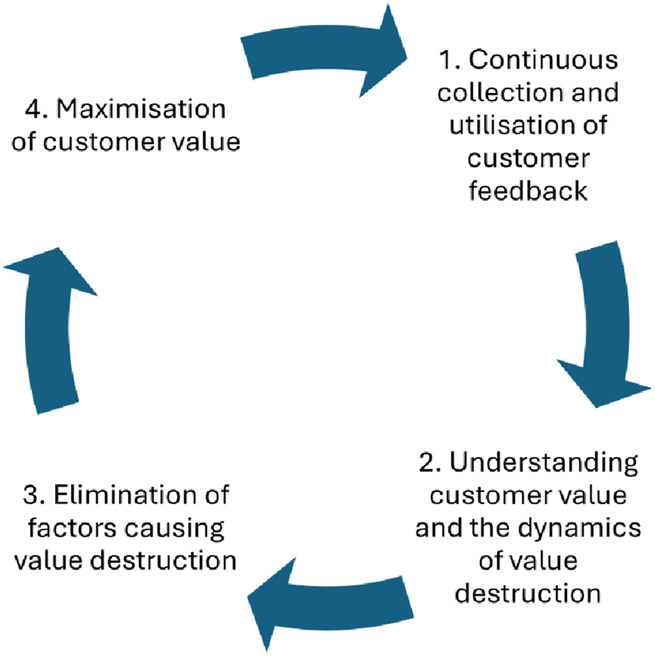
Continuous cycle for managing value in public healthcare services. Source: Author’s own work

The study also raises a broader societal concern. When basic services fail, value is not only lost in individual encounters. It undermines trust, social inclusion and, ultimately, the foundational promise of equality within the welfare state. To address this risk, public service organisations must systematically collect and act on customer feedback in order to identify, prevent and eliminate both value destruction and co-destruction in everyday operations. Figure 2 presents a proposed continuous improvement cycle designed to address these dynamics within public healthcare provision.
